# Acute bout of exercise downregulates thioredoxin‐interacting protein expression in rat contracting skeletal muscles

**DOI:** 10.14814/phy2.14388

**Published:** 2020-05-31

**Authors:** Song‐Gyu Ra, Emi Kawamoto, Keiichi Koshinaka, Maiko Iwabe, Yuki Tomiga, Hiroki Iizawa, Hiroki Honda, Yasuki Higaki, Kentaro Kawanaka

**Affiliations:** ^1^ Laboratory of Exercise Nutrition and Biochemistry Faculty of Sports and Health Science Fukuoka University Fukuoka Japan; ^2^ Fukuoka University Institute for Physical Activity Fukuoka Japan; ^3^ Department of Materials Engineering National Institute of Technology Nagaoka College Nagaoka Japan; ^4^ Department of Health and Sports Niigata University of Health and Welfare Niigata Japan; ^5^ Department of Nutrition Sapporo University of Health Sciences Sapporo Japan; ^6^ Laboratory of Exercise Physiology Faculty of Sports and Health Science Fukuoka University Fukuoka Japan

**Keywords:** AMP‐activated protein kinase, epitrochlearis, soleus, swimming, treadmill running

## Abstract

We previously reported that in rat skeletal muscle, disuse (i.e., decreased muscle contractile activity) rapidly increases thioredoxin‐interacting protein (TXNIP), which is implicated in the reduced glucose uptake. Accordingly, we sought herein to (a) determine the effect of exercise (i.e., increased muscle contractile activity) on muscle TXNIP protein expression, and (b) elucidate the mechanisms underlying the changes of TXNIP protein expression in response to exercise. Rat epitrochlearis and soleus muscles were dissected out after an acute bout of 3‐hr swimming (without weight loading) or 3‐hr treadmill running (15% grade at 9m/min). In a separate protocol, the isolated epitrochlearis and soleus muscles were incubated for 3 hr with AMP‐dependent protein kinase activator AICAR. Immediately after the cessation of the 3‐hr swimming, the TXNIP protein was decreased in epitrochlearis but not in soleus muscle. Conversely, 3‐hr treadmill running decreased the TXNIP protein in soleus but not in epitrochlearis muscle. TXNIP protein was decreased concomitantly with reduced postexercise muscle glycogen, showing that a decrease in TXNIP protein expression occurs in muscles that are recruited during exercise. In addition, 3‐hr incubation with AICAR decreased TXNIP protein in both isolated epitrochlearis and soleus muscles. Our results suggest that (a) an acute bout of exercise downregulates TXNIP protein expression in rat contracting skeletal muscles, and (b) the reduction in TXNIP protein expression in contracting muscles is probably mediated by AMPK activation, at least in part.

## Graphical abstract

Our present findings suggest that (1) increased muscle contractile activity acutely downregulates TXNIP protein expression in rat skeletal muscles, and (2) the reduction in TXNIP protein expression in contracting muscles is probably mediated by AMPK activation, at least in part.

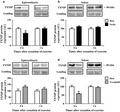

## 1 INTRODUCTION

Thioredoxin‐interacting protein (TXNIP), also known as vitamin D_3_ upregulated protein‐1 (VDUP‐1) or thioredoxin‐binding protein‐2 (TBP‐2), is a ubiquitously expressed protein. TXNIP was first identified as a protein that binds to and negatively regulates thioredoxin (Trx), a redox protein with important anti‐oxidative function (Chen & DeLuca, 1994; Junn et al., 2000; Nishiyama et al., 1999; Yamanaka et al., 2000). TXNIP can thereby modulate the cellular redox state and sensitize cells to oxidative stress (Chen & DeLuca, 1994; Junn et al., 2000; Nishiyama et al., 1999; Yamanaka et al., 2000). In addition, independent of its Trx‐binding property, TXNIP was more recently shown to be a key negative regulator of glucose uptake in peripheral tissues in both an insulin‐independent and an insulin‐dependent manner (Hui et al., 2008; Parikh et al., 2007; Waldhart et al., 2017; Wu et al., 2013; Yoshihara et al., 2010). Under fasting conditions, TXNIP suppresses glucose uptake directly by binding to the glucose transporters GLUT1 and GLUT4 and by inducing GLUTs endocytosis, in order to prevent hypoglycemia (Waldhart et al., 2017; Wu et al., 2013). In a postprandial state, insulin promotes the dissociation of TXNIP from GLUT4, which leads to the prevention of the endocytosis of GLUT4 (Waldhart et al., 2017). This consequently permits acute increases in the number of cell surface GLUT4 and glucose uptake in insulin‐sensitive tissues (Waldhart et al., 2017).

We reported that in rat soleus muscle, short‐duration (6‐hr) hindlimb immobilization resulted in increased TXNIP protein expression together with reductions in the insulin‐independent and insulin‐dependent glucose uptake (Kawamoto, Tamakoshi, Ra, Masuda, & Kawanaka, 2018). This result provided evidence that disuse, that is, decreased muscle contractile activity, rapidly increases the TXNIP protein expression and decreases glucose uptake in skeletal muscles. In contrast, a single bout of exercise, that is, increased muscle contractile activity, is well known to stimulate glucose uptake independently of insulin's action in skeletal muscles (Lee, Hansen, & Holloszy, 1995; Nesher, Karl, & Kipnis, 1985; Ploug, Galbo, & Richter, 1984; Yeh, Gulve, Rameh, & Birnbaum, 1995). This increase in glucose uptake is evident in rats during and immediately after exercise but reverses progressively, with little or no residual effects measured 3–4 hr after the cessation of exercise (Cartee et al., 1989; Wallberg‐Henriksson, Constable, Young, & Holloszy, 1988). As the effect of exercise on the insulin‐independent glucose uptake subsides, there is a substantial increase in the insulin‐stimulated glucose uptake in skeletal muscles (Garetto, Richter, Goodman, & Ruderman, 1984; Richter, Garetto, Goodman, & Ruderman, 1982; Wallberg‐Henriksson et al., 1988). It therefore seems reasonable to speculate that exercise downregulates the TXNIP protein expression in skeletal muscles, contributing to these increases in insulin‐independent and ‐dependent muscle glucose uptake.

Accordingly, the aim of this study was to determine whether an acute bout of exercise reduces muscle TXNIP protein expression. There is also considerable evidence that the activation of 5'‐adenosine monophosphate‐activated protein kinase (AMPK) mediates various exercise‐induced adaptations in skeletal muscles through an increase in AMP and decreases in phosphocreatine and ATP that occur in contracting muscles (Hardie & Sakamoto, 2006; Winder et al., 2000). We thus examined whether the activation of AMPK, which occurs in contracting muscles during exercise, downregulates the TXNIP protein expression in skeletal muscles. We also examined the effects of chronic exercise (i.e., training) on muscle TXNIP protein expression.

## 2 MATERIALS AND METHODS

### 2.1 Materials

Antibodies against TXNIP (#14715), Sirt3 (#5490), and acetyl CoA carboxylase (ACC, #3662) were purchased from Cell Signaling Technology. Anti‐thioredoxin reductase 2 (TrxR2) antibody (#PA1‐20940) was purchased from Thermo Fisher Scientific. Anti‐phospho ACC Ser^79^ antibody (#07‐303) was purchased from Millipore. Horseradish peroxidase (HRP)‐conjugated anti‐rabbit IgG was purchased from Vector Laboratories. Enhanced chemiluminescence reagent (ECL prime) was purchased from Amersham Biosciences. Ponceau S solution (#P7170‐L) and all other reagents were obtained from Sigma‐Aldrich.

### 2.2 Treatment of animals

The present research was approved by the Animal Care and Use Committee of Fukuoka University and by the Animal Studies Committee of Niigata University of Health and Welfare. Male Wistar rats were obtained from CLEA Japan. The animals were housed in individual cages controlled for temperature (23.5°C ± 0.7°C), humidity (34.0 ± 5.7%), and light (12 hr light‐dark cycle with lights on at 8 am). The animals were fed a standard rodent chow diet and water ad libitum.

### 2.3 Acute bout of swimming exercise protocol

Over 3 days before the experiment day, all rats were acclimated to swimming for 10 min. Rats (130–150 g) were divided into a resting control group (Rest) and a swimming group (Swim). Both groups of rats were fasted from 12:00 pm of the experiment day. Rats in the swimming group swam for 3 hr without any weight attached (Koshinaka, Kawasaki, Hokari, & Kawanaka, 2009; Koshinaka et al., 2008). The swimming rats finished swimming at 6:00 pm. After the exercise protocol, the rats were killed by cervical dislocation either immediately or 3 hr after the completion of the swimming. The resting control group was time‐matched with the swimming group, with the tissues of the resting control rats being collected at the same time as those of the swimming rats.

In the rats that were killed immediately after swimming, the epitrochlearis and soleus muscles were rapidly dissected and clamp‐frozen in liquid nitrogen for the subsequent glycogen assay, western blot analysis, or quantitative real‐time reverse transcription‐polymerase chain reaction (RT‐PCR) assay as described below. The rats to be killed 3 hr after the cessation of swimming were returned to their cages and remained fasting for another 3 hr. The epitrochlearis and soleus muscles were dissected out and used for the subsequent assays. The epitrochlearis and soleus muscles were chosen because they represent different fiber‐type compositions, as follows. Epitrochlearis: 15% type I, 20% type IIa, and 65% type IIb (Nesher, Karl, Kaiser, & Kipnis, 1980). Soleus: 84% type I, 16% type IIa, and 0% type IIb (Ariano, Armstrong, & Edgerton, 1973).

### 2.4 Acute bout of treadmill running exercise protocol

Three days before the experiment day, all rats were acclimated to treadmill running for 10 min. Rats (50–60 g) were divided into a resting control group (Rest) and a treadmill running group (Treadmill). Both groups of rats were fasted from 12:00 pm of the experiment day. Rats in the treadmill running group ran on a motorized treadmill up a 15% grade for 3 hr at 9 m/min (Iwabe, Kawamoto, Koshinaka, & Kawanaka, 2014). The treadmill running rats finished running at 6:00 pm. Following the exercise protocol, the rats were killed by cervical dislocation either immediately or 3 hr after the completion of the treadmill running. The resting control group was time‐matched with the treadmill running group, with the tissues of the resting control rats being collected at the same time as those of the swimming rats.

In the rats that were killed immediately after treadmill running, the epitrochlearis and soleus muscles were rapidly dissected and clamp‐frozen in liquid nitrogen for the subsequent glycogen assay, western blot analysis, or RT‐PCR assay as described below. The rats to be killed 3 hr after the cessation of treadmill running were returned to their cages and remained fasting for another 3 hr. The epitrochlearis and soleus muscles were dissected out and used for the subsequent assays.

### 2.5 Isolated muscle incubation protocol

In a separate protocol, to examine the effects of AMPK activation, we used 5‐aminoimidazole‐4‐carboxamide 1‐β‐D‐ribofuranoside (AICAR), an analog of AMP that is capable of stimulating AMPK activity. The epitrochlearis and soleus muscles were removed from sedentary rats (130–150 g) under inhalational anesthesia with isoflurane. The isolated muscles were incubated as described (Kawanaka, Han, Gao, Nolte, & Holloszy, 2001). Briefly, epitrochlearis and soleus muscles were placed in 4 ml of oxygenated Krebs–Henseleit bicarbonate buffer in the presence of 8 mM glucose, 32 mM mannitol, and 0.1% radioimmunoassay (RIA)‐grade bovine serum albumin (BSA) with or without one of two concentrations (0.5 mM or 2.0 mM) of AICAR. The muscles were incubated for 3 hr with shaking at 35°C, and the flasks were gassed continuously with 95% O_2_, 5% CO_2_. During a 3‐hr incubation, the incubation medium was replaced with fresh incubation medium at 1 hr after the start of incubation. Immediately after the cessation of the 3‐hr incubation, the epitrochlearis and soleus muscles were clamp‐frozen and stored at −80°C for the subsequent western blot analysis and RT‐PCR assay.

### 2.6 Chronic treadmill running training protocol

Rats (130–150 g) were divided into an untrained sedentary control group (Sedentary control) and a treadmill running training group (Treadmill training). The rats of the latter group were trained on a motorized treadmill without a grade for a total of 4 weeks. The speed and duration of treadmill running were increased to 25 m/min and 60 min/day, respectively, during the first week, and they were then made to exercise at 25 m/min for 60 min, 5 days/week for 3 weeks. All rats were maintained in individual cages and fed a standard rodent chow diet and water ad libitum.

Approximately 24 hr after the last bout of exercise, the rats were anesthetized with isoflurane, and their forelimb triceps and hindlimb soleus muscles were dissected out and clamp‐frozen in liquid nitrogen for a western blot analysis. The triceps muscle was chosen because the functional characteristic and fiber‐type composition of this muscle is very similar to those of the epitrochlearis muscle (Matsumoto, Nagatomo, Mori, Ohira, & Ishihara, 2007). In addition, the rat triceps and epitrochlearis muscles show a similar adaptive response to exercise training (Host, Hansen, Nolte, Chen, & Holloszy, 1998). We took samples of muscle approx. 24 hr after the last bout of exercise, because this length of time is long enough to permit the acute effects of exercise on insulin‐dependent muscle glucose uptake to wear off (Cartee et al., 1989). All rats were fasted for ~16 hr before the muscle sampling.

### 2.7 Skeletal muscle glycogen concentration

The epitrochlearis and soleus muscles were weighed and homogenized with 0.3 M perchloric acid, and extracts were assayed for glycogen by the amyloglucosidase method (Passonneau & Lauderdale, 1974).

### 2.8 Western blot analysis

The frozen epitrochlearis and soleus muscles were homogenized in ice‐cold RIPA buffer (FujiFilm Wako Pure Chemical Corp.) containing protease inhibitor cocktail (ProteoGuard™ Protease Inhibitor Cocktail containing 0.8 μM aprotinin, 50 μM bestatin, 20 μM leupeptin, 10 μM pepstatin A, 1 mM PMSF; Takara) and phosphatase inhibitor cocktail (Nacalai Tesque). The homogenates were then rotated end‐over‐end for 60 min at 4°C and centrifuged at 10,000*g* for 15 min at 4°C.

Aliquots of the supernatants were treated with Laemmli sample buffer containing 100 mM dithiothreitol (BioRad). Protein levels were quantified by a bicinchoninic acid (BCA) assay (Pierce™ BCA Protein Assay Kit, Thermo Fisher Scientific). For the measurement of TXNIP, Sirt3 and TrxR2, equal amounts of total protein (20 µg) were electrophoresed by 10% sodium dodecyl sulfate‐polyacrylamide gel electrophoresis (SDS‐PAGE). For the measurement of phospho‐ACC and total ACC, total protein (20 µg) were electrophoresed by 5% SDS‐PAGE. The resolved proteins were then transferred to a polyvinylidene difluoride membrane and blocked with 5% fat‐free skim milk in Tris‐buffered saline containing 0.1% Tween‐20 (TBST), pH 7.5. After blocking for 60 min at room temperature (RT), the membranes were washed in TBST and incubated overnight with the appropriate primary antibody at 4°C.

After the membranes were washed, the membranes were further incubated with HRP‐conjugated anti‐rabbit IgG for 60 min at RT. Bound antibody was detected by ECL prime Western Blotting Detection Reagent and analyzed using an Amersham Imager 600 (GE Healthcare Life Sciences, Tokyo). Equal protein concentrations were loaded in each lane and also confirmed by Ponceau S staining of the blot membrane.

### 2.9 Real‐time PCR analysis

Total RNA was extracted from epitrochlearis and soleus muscles using the FastGene™ RNA Basic Kit (Nippon Genetics) according to the manufacturer's instructions. The RNA concentrations and purity were measured using a NanoDrop™ Lite spectrophotometer (Thermo Fisher Scientific). Total RNA was reverse transcribed with PrimeScript RT Master Mix (Takara). Synthesized cDNA was used as a template for the qPCR reaction using PowerUp SYBR Green Master Mix probes to analyze TXNIP mRNA levels with the Step One Real Time PCR system (Applied Biosystems).

The primer sequences were as follows: β‐actin, forward, 5′‐GGAGATTACTGCCCTGGCTCCTA‐3′, reverse, 5′‐GACTCATCGTACTCCTGCTTGCTG‐3′; TXNIP, forward, 5′‐GGCAATCAGTAGGCAAGTCTCCA‐3′, reverse, 5′‐GTTCCGACATTCACCCAGCA‐3′. The expression level of TXNIP gene was normalized against the corresponding amount of β‐actin mRNA. The relative amounts of each product were calculated using the comparative Ct method.

### 2.10 Statistical analysis

All data are expressed as mean ± *SE*. The normality of the data was determined by the Shapiro‐Wilk test. Because the data obtained were normally distributed, we used the unpaired sample *t*‐test to analyze differences between pairs of groups and a one‐way analysis of variance (ANOVA) with Bonferroni's post‐hoc comparison to analyze data sets of more than two groups. We set the significance level at *p < *.05 and used GraphPad Prism 6 software (GraphPad) for all data analyses.

## 3 RESULTS

### 3.1 The skeletal muscle glycogen concentration immediately after swimming or treadmill running

**FIGURE 1:**
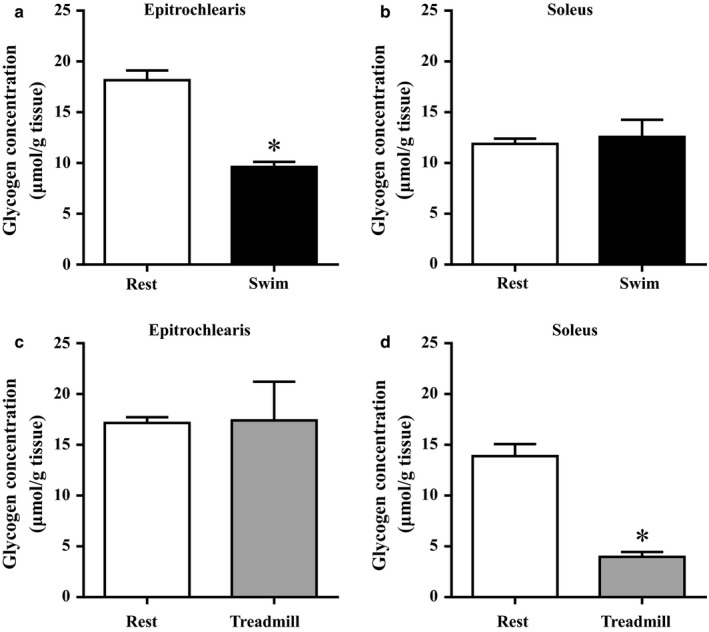
Glycogen concentration in rat epitrochlearis and soleus muscles immediately after an acute bout of swimming and running. Rats were subjected to an acute 3‐hr‐long bout of swimming without a weight (Swim). Immediately after the cessation of swimming, epitrochlearis (a) and soleus (b) muscles were dissected out and used for the measurement of glycogen concentration. Other rats were subjected to an acute 3‐hr‐long bout of treadmill running up a 15% grade at 9 m/min (Treadmill). Immediately after the cessation of treadmill running, epitrochlearis (c) and soleus (d) muscles were dissected out and used for the measurement of glycogen concentration. The time‐matched resting control (Rest) rats started fasting when the exercised rats started fasting (3 hr before starting exercise). Values are mean ± *SE*; *n* = 5–8 muscles. *p < .05 versus time‐matched resting control muscles

We measured the muscle glycogen concentration in the rat skeletal muscles immediately after an acute bout of swimming or treadmill running (Figure 1). Swimming reduced the muscle glycogen concentration in epitrochlearis muscle by 47% compared to the time‐matched resting control (*p* < .05), but no significant difference in muscle glycogen concentration was found in soleus muscle between the resting control and swimming groups. No significant difference in the muscle glycogen concentration was found in epitrochlearis muscle between the resting control and treadmill running groups. However, treadmill running significantly reduced the muscle glycogen concentration in soleus muscle by 72% compared to the time‐matched resting controls (*p < *.05). These glycogen reduction pattern in different skeletal muscles are supported by previous studies showing that, in the rat, the fast‐twitch forelimb muscles (e.g., epitrochlearis muscle) are more heavily recruited than the slow‐twitch hindlimb antigravity muscles (e.g., soleus muscle) during swimming, whereas the opposite is the case during treadmill running (Roy, Hutchison, Pierotti, Hodgson, & Edgerton, 1991; Sullivan & Armstrong, 1978; Terada & Tabata, 2004).

### 3.2 The ACC phosphorylation immediately after swimming or treadmill running

We measured the ACC phosphorylation in skeletal muscles immediately after rats underwent an acute bout of swimming or treadmill running. Since active AMPK phosphorylates ACC at the Ser^79^ site, ACC phosphorylation is considered an intracellular reporter of AMPK activity in vivo. Swimming significantly increased the ACC phosphorylation compared to the time‐matched resting control group in the epitrochlearis muscle (Rest: 100 ± 12 arbitrary units [AU], *n* = 6; Swim: 197 ± 14 AU, *n* = 6, *p* < .05), but not in the soleus muscle (Rest: 100 ± 6 AU, *n* = 4; Swim: 76 ± 16 AU, *n* = 4). There was no significant change in ACC phosphorylation in epitrochlearis muscle after treadmill running (Rest: 100 ± 12 AU, *n* = 6; Treadmill: 91 ± 2 AU, *n* = 6). However, treadmill running significantly increased the ACC phosphorylation in soleus muscle compared to the time‐matched resting control (Rest: 100 ± 12 AU, *n* = 7; Treadmill: 280 ± 8 AU, *n* = 6, *p < *.05). Neither swimming nor treadmill running affected the total ACC in the epitrochlearis or soleus muscles.

### 3.3 The TXNIP protein expression after acute bout of swimming or treadmill running

**FIGURE 2:**
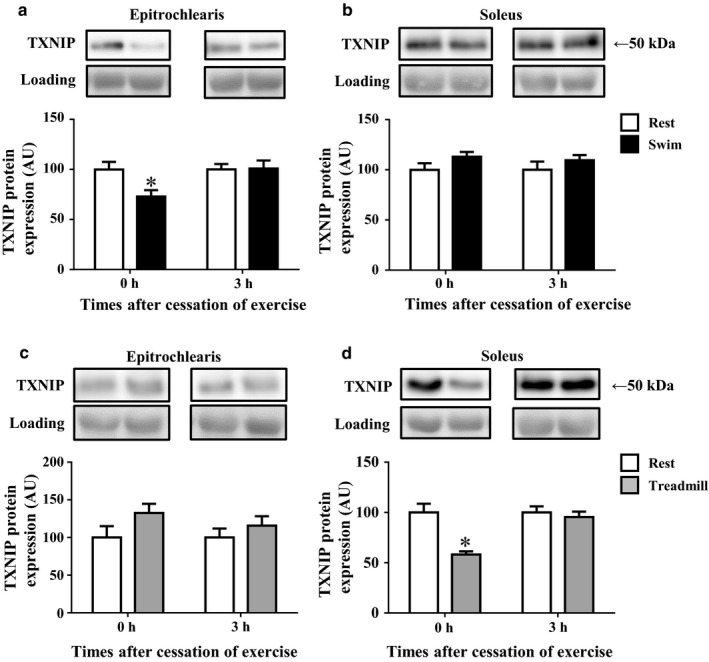
The TXNIP protein expression in rat epitrochlearis and soleus muscles after swimming or treadmill running. Rats were subjected to an acute 3‐hr‐long bout of swimming without a weight (Swim). Immediately (0 hr) or 3 hr after the cessation of swimming, epitrochlearis (a) and soleus (b) muscles were dissected out and used for the measurement of TXNIP protein. Other rats were subjected to an acute 3‐hr‐long bout of treadmill running up a 15% grade at 9 m/min (Treadmill). Immediately (0 hr) or 3 hr after the cessation of treadmill running, epitrochlearis (c) and soleus (d) muscles were dissected out and used for the measurement of TXNIP protein. The time‐matched resting control (Rest) rats started fasting when the exercised rats started fasting (3 hr before starting exercise). Values are mean ± *SE*; *n* = 9 (a), *n* = 8–9 (b), *n* = 7–9 (c), *n* = 7–9 (d). *p < .05 versus the time‐matched resting control group

As shown in Figure 2a, the acute bout of swimming significantly reduced the TXNIP protein expression in the rats' epitrochlearis muscle immediately after the cessation of exercise by 27% relative to the time‐matched resting control (*p < *.05). The decreased TXNIP protein expression in the epitrochlearis muscles had returned to the time‐matched resting control level at 3 hr after the cessation of exercise (Figure 2a). In contrast, swimming did not affect the expression of TXNIP protein in soleus muscle immediately or at 3 hr after the cessation of exercise (Figure 2b).

As shown in Figure 2c, the acute bout of treadmill running did not alter the expression of TXNIP protein in epitrochlearis muscle, whereas treadmill running significantly reduced the TXNIP protein expression in soleus muscle immediately after the cessation of running exercise by 42% relative to the time‐matched resting control (*p* < .05, Figure 2d). The decreased expression of TXNIP protein in the soleus muscles had returned to the time‐matched resting control level at 3 hr after the cessation of the exercise (Figure 2d).

### 3.4 The TXNIP mRNA expression after acute bout of swimming or treadmill running

**FIGURE 3:**
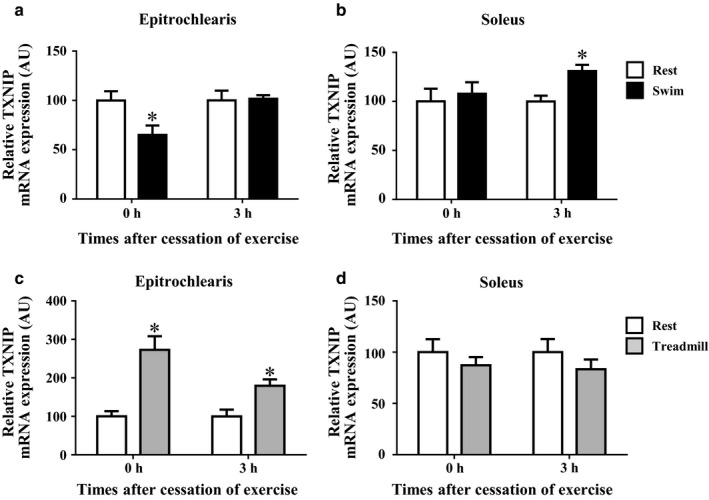
The TXNIP mRNA expression in rat epitrochlearis and soleus muscles after swimming or treadmill running. Rats were subjected to an acute 3‐hr‐long bout of swimming without a weight (Swim). Immediately (0 hr) or 3 hr after the cessation of swimming, epitrochlearis (a) and soleus (b) muscles were dissected out and used for the measurement of TXNIP mRNA. Other rats were subjected to an acute 3‐hr‐long bout of treadmill running up a 15% grade at 9 m/min (Treadmill). Immediately (0 hr) or 3 hr after the cessation of treadmill running, epitrochlearis (c) and soleus (d) muscles were dissected out and used for the measurement of TXNIP mRNA. The time‐matched resting control (Rest) rats started fasting when the exercised rats started fasting (3 hr before starting exercise). Values are mean ± *SE*; *n* = 8–9 (a), *n* = 9 (b), *n* = 7–9 (c), *n* = 7–9 (d). *p < .05 versus the time‐matched resting control group

As shown in Figure 3a, the acute bout of swimming significantly reduced the TXNIP mRNA expression in the rats' epitrochlearis muscle immediately after the cessation of exercise by 35% compared to the time‐matched resting control (*p < *.05). The decreased TXNIP mRNA expression in the epitrochlearis muscles had returned to the time‐matched resting control level at 3 hr after the cessation of exercise (Figure 3a). In contrast, the TXNIP mRNA expression in soleus muscle was not changed immediately after the cessation of swimming, but it was increased at 3 hr after the swimming by 31% relative to the time‐matched resting control group (*p* < .05, Figure 3b).

As shown in Figure 3c, the TXNIP mRNA expression in epitrochlearis muscle was increased immediately and at 3 hr after the cessation of treadmill running by 173% and 79%, respectively, compared to the time‐matched resting control group, whereas the treadmill running did not affect the TXNIP mRNA expression in soleus muscle (*p* < .05, Figure 3d).

### 3.5 The TrxR2 protein expression after acute bout of swimming or treadmill running

**FIGURE 4:**
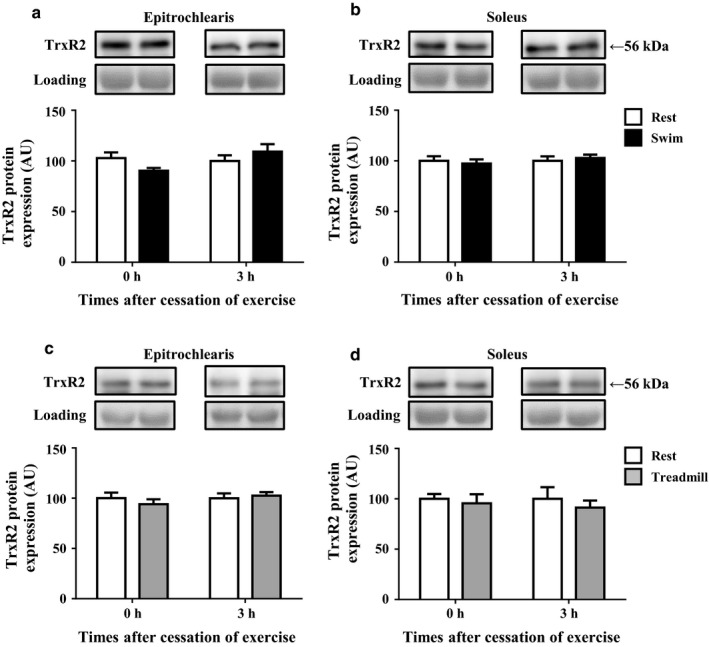
The TrxR2 protein expression in rat epitrochlearis and soleus muscles immediately after swimming or treadmill running. Rats were subjected to an acute 3‐hr‐long bout of swimming (Swim). Immediately (0 hr) or 3 hr after the cessation of swimming, epitrochlearis (a) and soleus (b) muscles were dissected out and used for the measurement of TrxR2 protein. Other rats were subjected to an acute 3‐hr‐long bout of treadmill running (Treadmill). Immediately (0 hr) or 3 hr after the cessation of treadmill running, epitrochlearis (c) soleus (d) muscles were dissected out and used for the measurement of TrxR2 protein. The time‐matched resting control (Rest) rats started fasting when the exercised rats started fasting (3 hr before starting exercise). Values are mean ± *SE*; *n* = 9 (a), *n* = 9 (b), *n* = 7–9 (c), *n* = 7–9 (d)

As shown in Figure 4, the acute bouts of swimming and treadmill running did not alter the expression of TrxR2 protein in epitrochlearis or soleus muscle.

### 3.6 The effects of AICAR administration on the TXNIP protein and mRNA expression

**FIGURE 5:**
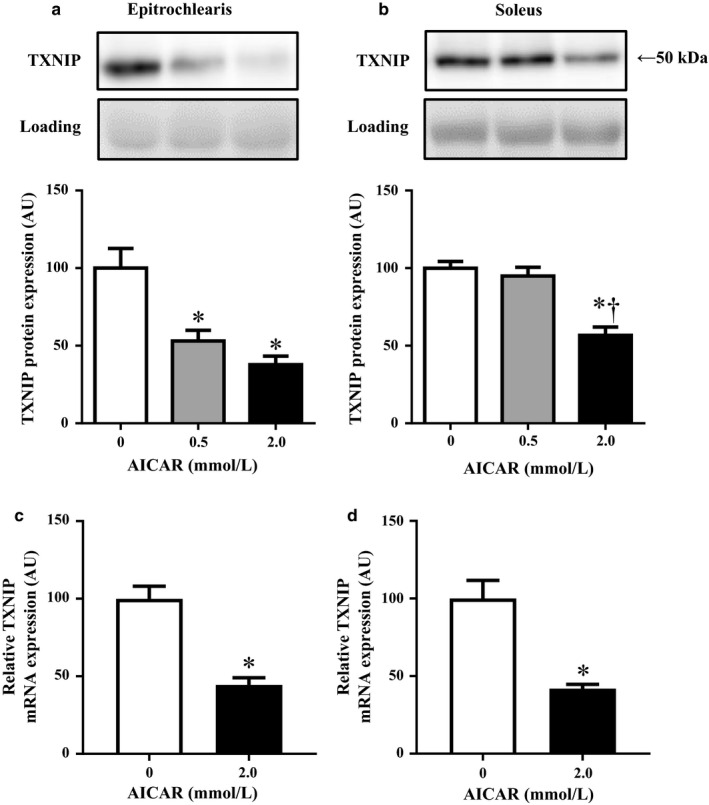
The TXNIP protein and mRNA expression in rat epitrochlearis and soleus muscles after 3‐hr incubation with 0.5 mM or 2.0 mM AICAR. The epitrochlearis and soleus muscles were removed from sedentary rats. The isolated muscles were incubated in medium containing 0 mM, 0.5 mM, or 2.0 mM of AICAR for 3 hr with shaking at 35°C, and the flasks were gassed continuously with 95% O_2_, 5% CO_2_. Immediately after the cessation of the 3‐hr incubation, the epitrochlearis (a and c) and soleus (b and d) muscles were clamp‐frozen and used for the measurement of TXNIP protein and mRNA. Values are mean ± *SE*; *n* = 8 (a), *n* = 8 (b), *n* = 8 (c), *n* = 8 (d). *p < .05 versus 0 mM AICAR. ^†^ *p* < .05 versus 0.5 mM AICAR

As shown in Figure 5a, the incubation of rat epitrochlearis muscle for 3 hr with 0.5 mM or 2.0 mM AICAR induced 47% and 62% reductions in TXNIP protein expression, respectively, compared to the corresponding muscles incubated with 0 mM AICAR (*p < *.05). The incubation of rat soleus muscle for 3 hr with 2.0 mM AICAR caused a 43% reduction in TXNIP protein expression compared to the corresponding muscle incubated with 0 mM AICAR (*p < *.05), whereas the incubation of soleus with 0.5 mM AICAR did not affect the TXNIP protein expression (Figure 5b). As shown in Figure 5c and d, the incubation of rat epitrochlearis and soleus muscle for 3 hr with 2.0 mM AICAR induced 56% and 58% reductions in the expression of TXNIP mRNA, respectively compared to the corresponding muscles incubated with 0 mM AICAR (*p* < .05). The 0.5 mM and 2.0 mM AICAR incubation for 3 hr did not alter the TrxR2 protein expression in the epitrochlearis muscle or the soleus muscle (data not shown)**.**

### 3.7 The effects of chronic 4‐week treadmill running training on the expressions of Sirt3 and TXNIP protein

To examine the cumulative effects of chronic exercise (i.e., training), we measured the Sirt3 and TXNIP protein expressions in skeletal muscles after the rats underwent 4 weeks of treadmill running training. Skeletal muscles were dissected out 24 hr after the last bout of exercise for the examination of the resting baseline protein expression level. Compared to the sedentary control group, the rats that underwent 4 weeks of treadmill running training showed significantly increased Sirt3 protein expression in the soleus muscle (Sedentary control: 100 ± 2 AU, *n* = 11; Treadmill trained: 135 ± 6 AU, *n* = 11, *p* < .05), but not in the triceps muscle (Sedentary control: 100 ± 6 AU, *n* = 11; Treadmill trained: 102 ± 5 AU, *n* = 11). This result indicates that the soleus but not triceps muscles are fully recruited during treadmill running exercise, since local muscle contractile activity regulates the Sirt3 protein expression in skeletal muscles (Hokari et al., 2010).

However, despite the increase in Sirt3 in the trained soleus muscles, the treadmill running training did not alter the expression of TXNIP protein in soleus muscle (Sedentary control: 100 ± 8 AU, *n* = 11; Treadmill trained: 93 ± 6 AU, *n* = 11, *p* = .50). Similarly, the treadmill running training did not alter the expression of TXNIP protein in triceps muscle (Sedentary control: 100 ± 11 AU, *n* = 11; Treadmill trained: 99 ± 9 AU, *n* = 11, *p* = .96). Thus, although an acute bout of treadmill running decreased TXNIP protein in the exercised soleus muscle immediately after the cessation of running (Figure 2d), the chronic 4‐week treadmill running did not alter the resting baseline expression of TXNIP protein in the trained soleus muscles.

## 4 DISCUSSION

We observed that the TXNIP protein expression was decreased in the rats' forelimb epitrochlearis muscle but not in the hindlimb soleus muscle immediately after the cessation of an acute bout of swimming (Figure 2a and b). In contrast, the acute bout of treadmill running caused a decrease in TXNIP protein in the soleus muscle but not in the epitrochlearis muscle isolated immediately after exercise (Figure 2c and d). Since the TXNIP protein expression was decreased in muscles in which the muscle glycogen levels were reduced during exercise (Figure 1), our results suggest that exercise probably downregulates the TXNIP protein expression in the muscle fibers that are recruited during exercise.

Although it is not yet known how muscle senses specifically "exercise signals" to reduce the TXNIP protein expression, TXNIP was reported to be up‐regulated by glucose in pancreatic beta cells (Shalev et al., 2002). Since prolonged exercise decreases the blood glucose level, we initially hypothesized that the exercise‐induced reduction in the skeletal muscles' TXNIP protein expression is due to changes in the blood glucose level. However, this seems unlikely. Our present experiments demonstrated that exercise downregulated the TXNIP protein expression in contracting muscles during exercise. Thus, the exercise‐induced reduction in TXNIP protein expression is due mainly to local factors that are intrinsic to the muscles involved in the exercise rather than by systemic factors such as the blood glucose level.

To investigate the cellular mechanism by which local muscle contractile activity regulates the TXNIP protein expression, we examined the effect of the pharmacological AMPK activator AICAR on the TXNIP protein expression. We used AICAR because the activation of AMPK was suggested to be involved in exercise‐induced local adaptations (such as the increases in GLUT4, peroxisome proliferator‐activated receptor‐γ coactivator‐1α [PGC‐1α], and mitochondrial biogenesis) in skeletal muscle through an increase in AMP and decreases in phosphocreatine and ATP that occur in contracting muscles (Holmes, Kurth‐Kraczek, & Winder, 1999; Ojuka, Nolte, & Holloszy, 2000; Terada & Tabata, 2004; Winder et al., 2000). Our present findings revealed that the TXNIP protein expression was decreased in both the epitrochlearis and soleus muscles which were isolated and then incubated for 3 hr with AICAR (Figure 5a and b). Our findings also demonstrated that swimming and treadmill running exercise increased ACC phosphorylation, an indicator of the in vivo AMPK activation level, in muscles recruited during exercise (see the Results section). Taken together, our results suggest that the decreased TXNIP protein expression in exercised muscles is probably mediated by AMPK activation, at least in part.

Wu et al. observed that the activation of AMPK resulted in the phosphorylation and degradation of TXNIP in rat hepatocytes without a decrease in TXNIP mRNA, showing the possibility that an AMPK‐dependent phosphorylation of TXNIP leads to its accelerated degradation (Wu et al., 2013). In our present study, the AICAR‐induced decrease in TXNIP protein was accompanied by reduced mRNA expression in both fast‐twitch epitrochlearis muscles and slow‐twitch soleus muscles (Figure 5c and d); this results is not consistent with the finding obtained in the Wu et al. study (2013). It is thus possible that in skeletal muscles, AMPK activation both promotes TXNIP protein degradation and represses the synthesis of TXNIP protein at the transcription level, leading to the decrease in TXNIP protein.

TXNIP binds to and facilitates GLUT4 endocytosis in the absence of insulin in 3T3‐L1 adipocytes (Waldhart et al., 2017). Thus, in insulin‐sensitive tissues (i.e., skeletal muscle and adipose tissue), TXNIP seems to decrease the number of GLUT4 transporters on the cell surface and reduce the glucose uptake in order to prevent hypoglycemia under fasting conditions. Therefore, in our present study, the exercise‐induced TXNIP downregulation may have relieved the TXNIP‐facilitated GLUT4 endocytosis, promoting the insulin‐independent increase in the cell surface GLUT4 number and the glucose uptake in the contracting muscles during and after exercise. This possibility can be supported by our previous studies showing that an acute 3‐hr bout of swimming or treadmill running— the exercise modes used in the present study — increases glucose uptake in the contracting muscles independently of insulin action immediately after the cessation of exercise (Iwabe et al., 2014; Koshinaka et al., 2008, 2009). However, the evidence obtained in our research is not sufficient to conclude that there is a causal relationship between TXNIP downregulation and increased insulin‐independent glucose uptake in exercised muscle. Further investigations are required to elucidate any causal relationship.

Although TXNIP functions as an adaptor for the basal GLUT4 endocytosis in order to prevent hypoglycemia under fasting conditions, insulin releases TXNIP from GLUT4 and prevents GLUT4 from being endocytosed in the postprandial state (Waldhart et al., 2017). These events acutely increases the cell surface GLUT4 number and glucose uptake in insulin‐sensitive tissues (i.e., skeletal muscle and adipose tissue) (Waldhart et al., 2017). In our previous studies, an acute 3‐hr bout of swimming or treadmill running exercise as used in the present study increased the insulin sensitivity for glucose uptake in skeletal muscles 2–4 hr after the cessation of exercise (Iwabe et al., 2014; Koshinaka et al., 2008, 2009). Thus, the exercise‐induced downregulation of TXNIP that we observed herein might be related to this postexercise increase in muscle insulin sensitivity for glucose uptake. However, this possibility is unlikely, since the time course of the changes in TXNIP protein expression after the cessation of exercise is not consistent with the changes in insulin sensitivity. That is to say, in the present study, although the TXNIP protein expression was decreased immediately after exercise, it returned to the resting baseline level in the muscles with high insulin sensitivity at 3 hr after the cessation of exercise (Figure 2a and d). Thus, TXNIP downregulation is not likely to be involved in the postexercise increase in muscle insulin sensitivity.

The generation of reactive oxygen species (ROS) occurs mainly in the mitochondria of contracting skeletal muscles during exercise, leading to the induction of oxidative stress in exercised muscles. On the other hand, thioredoxin (Trx) plays a pivotal role in scavenging abnormal ROS and contributes to the defense against oxidative stress (Kondo, Nakamura, Masutani, & Yodoi, 2006). TXNIP binds to and negatively regulates Trx, whereas thioredoxin reductase (TrxR) reduces and activates Trx (Biaglow & Miller, 2005; Lee, Kim, & Lee, 2013). We thus hypothesized that the combination of both TXNIP downregulation and TrxR upregulation prevents oxidative stress and plays a role in the cellular antioxidant defense system in skeletal muscles during and after an acute bout of exercise. However, in our present study, the acute bout of exercise (both swimming and treadmill running) did not increase the protein expression of TrxR2, which is localized mainly in the mitochondria, in exercised muscle (Figure 4). Thus, TXNIP downregulation, but not TrxR2 upregulation, seems to play some role in the cellular antioxidant defense system in exercised muscles during and after exercise.

In conclusion, our present findings demonstrated for the first time that an acute bout of exercise (both swimming and treadmill running) results in decreased TXNIP protein expression in the contracting muscles of rats during exercise (Figure 2a and d). The exercise‐induced TXNIP downregulation may promote the insulin‐independent increase in glucose uptake in the contracting muscles during exercise. Our results also demonstrated that the pharmacological activation of AMPK downregulates the skeletal muscle TXNIP protein expression in a dose‐dependent manner. These findings suggest that the decrease in TXNIP protein expression in contracting muscles is likely to be mediated by AMPK activation, at least in part. Since decreased TXNIP protein expression rapidly returns to resting levels within 3 hr after the cessation of an acute bout of exercise, there is no cumulative effect of chronic exercise (i.e., training) on TXNIP protein expression.

## CONFLICT OF INTEREST

The authors have no conflicts of interest.
